# Self-Rated Oral Health and Associated Factors among an Adult Population in Rural India—An Epidemiological Study

**DOI:** 10.3390/ijerph18126414

**Published:** 2021-06-13

**Authors:** Meghashyam Bhat, Sreevidya Bhat, Kaye Frances Roberts-Thomson, Loc Giang Do

**Affiliations:** 1 Australian Research Centre for Population Oral Health, The University of Adelaide, Adelaide 5005, Australia; kaye.robertsthomson@adelaide.edu.au; 2 Srinivas Institute of Dental Sciences, Mangalore and Affiliated to Rajiv Gandhi University of Health Sciences, Bengaluru 560041, Karnataka, India; ksvidya5@yahoo.co.in; 3 School of Dentistry, The University of Queensland, Brisbane 4006, Australia; l.do@uq.edu.au

**Keywords:** self-rated oral health, rural, adults, DMFT, periodontal disease, dental visiting

## Abstract

Background: To determine the perception of oral health status and its associated factors among adults living in rural areas in Karnataka state, India. Methods: A cross-sectional study was conducted among adults in the age group of 35–54 years old residing in villages in a southern state in India. The main outcome measure was poor self-rated oral health (SROH) among adults in rural India. Results: About 873 adults participated in the study. The prevalence of poor SROH was 15.2%. Adults of age 40–44 years, females, those in lower socioeconomic conditions, and those with high caries experience (DMFT ≥ 4) and periodontal disease were associated with poor SROH. Those who had visited a dentist in the previous one year were 1.9 times more likely to report poor oral health. Conclusions: Nearly 15% of rural people reported poor oral health. Socioeconomic conditions, sex, age, smoking, and dental visiting were associated with poor SROH. People’s perception of poor oral health was associated with severe periodontitis and DMFT ≥ 4. A dose–response relationship was observed between experience with dental caries and poor SROH.

## 1. Introduction

Oral health is an important indicator of overall health and is multifaceted. In epidemiological research, oral health is measured mainly through clinical examination for dental caries, periodontal disease, tooth loss, and unmet dental treatment needs. Some studies have used a single item multidimensional global rating scale, ‘self-rated oral health’ (SROH), measured using an ordinal scale with five points varying from ‘excellent’ to ‘poor’ in order to capture the multiple aspects of oral health [1]. The self-rating of oral health is an economical health assessment method when used in population studies.

SROH has been shown to be linked to many factors. It provides a subjective experience of the psychosocial oral health outcomes [2]. Furthermore, SROH has shown an association with clinical oral health outcomes and other correlates such as psychosocial factors, dental care seeking behavior, and perceived stress [3]. Previous research has shown that poor SROH has also shown to be a predictor of five- and ten-year tooth loss [4]. Perceived oral health status has been associated with the clinical conditions (such as number of teeth present in the mouth, dental caries, missing teeth, periodontal pockets) [5,6,7], oral symptoms (such as toothache, gingival bleeding, difficulty in speaking due to dental problems), and status of denture [2,8,9]. Similar to clinical oral health outcomes, SROH has demonstrated socioeconomic inequality [9,10,11].

Studies have shown that it is important to use clinical data along with SROH measures [12]. Although oral examinations are considered the gold standard to assess oral health status [13], SROH is a valid method for epidemiological data collection [14,15]. Perceptions of oral health vary among ethnic groups and people of different cultural backgrounds [5]. Most of the published research involving SROH was from developed countries and some from developing countries [2,5,9]. India is a developing country and nearly 70% of the population reside in rural areas. The burden of oral diseases is high with significant impact on the quality of life of individuals [16,17,18]. Furthermore, most of the dentists are located in urban areas leading to the inverse care law. Due to various barriers to oral health care seeking rural people in India visit dentists only when there is a problem that affects their daily routine. In such a scenario, it is essential to understand people’s perception about their oral health. Most of the oral health research in India has been conducted by employing clinical measures. There is no published research which has investigated people’s perception of oral health and associated factors in rural India. Hence, the study was conducted to determine the perception of oral health status and its associations with socioeconomic status, health behavior, and clinical oral conditions among adults living in the rural areas of Karnataka state, India.

## 2. Material and Methods

Data collected from a larger population-based study ‘Factors associated with periodontal diseases and oral health related quality of life of rural people in India’ was utilised for this study [17,18]. The data were collected according to the Australian National Survey of Adult Oral Health guidelines [19]. The study protocol was in accordance with the Declaration of Helsinki (2008) and followed the STROBE guidelines. The sample size for the main study was calculated using the CDC-Epi-info TM 6 software to detect a 25% difference in the prevalence of periodontitis between the two communities based on the assumption that the prevalence of periodontitis to be nearly 40% in the coastal districts [20]. Expecting a refusal rate of 25%, the final sample size was estimated to be 1160. A multistage stratified cluster random sampling was followed. Two of the three coastal districts of Karnataka state were selected, and in the next step, five sub-provinces (taluks) of the coastal districts were selected. This was followed by the random selection of 50 villages from five sub-provinces and participants in the age group of 35–54 years old, selected randomly from households that were chosen following a skip pattern. Written informed consent was obtained prior to a face-to-face interview and oral examination. Details regarding the development and pilot testing of the questionnaire as well as training and calibration of examiner can be found elsewhere [17]. Data on sociodemographic details, oral health behaviour, and self-rated oral health were obtained through interview. Data on SROH were captured by asking the close-ended question, “How would you rate your oral health?” and the responses recorded were Poor/Fair/Good/Very Good/Excellent. Caries experienced (measured by using decayed, missing, and filled teeth (DMFT)) and periodontal status obtained through oral examination were included in this study. Oral examination was performed using disposable plain mouth mirrors and a PCP2 probe under artificial lighting (using a powerful head lamp).

This study is an extension of previous research^17^ in which the authors investigated poor self-rated oral health, a subjective measure, as the main outcome. The explanatory variables were DMFT, periodontitis, age, gender, education, monthly per capita income, material circumstances, status of tobacco users, and oral health behaviour. Periodontal disease was categorised into ‘No/Mild’, ‘Moderate’, and ‘Severe’ based on the case-definition given by CDC-AAP and updated by Eke, Page [21,22]. According to case definition, the ‘Mild’ category included people with ≥2 interproximal sites with CAL ≥ 3 mm, ≥2 interproximal sites with a pocket depth ≥ 4 mm (not on same tooth), or one site with a pocket depth ≥ 5 mm. The ‘Moderate’ category included: ≥2 interproximal sites with CAL ≥ 4 mm (not on the same tooth) or ≥2 interproximal sites with a pocket depth ≥ 5 mm (not on the same tooth). The ‘Severe’ category included: ≥2 interproximal sites with CAL ≥ 6 mm (not on same tooth) and ≥1 interproximal site with a pocket depth ≥5 mm. DMFT was calculated by summing decayed, filled, and missing teeth, and later categorised as quartiles. Age was categorised as 35–39, 40–44, 45–49, and 50–54 years, and education was categorised into primary/less, secondary, and post-secondary. Per capita income was used to determine the socioeconomic status (SES) [23], which was categorised as upper, upper-middle, middle, lower middle, and low income groups. Material circumstances were used as a socioeconomic indicator and was calculated based on the type of housing, house ownership, housing density, and vehicle ownership. Each of the items were given scores of 1 or 0 that was summed up and dichotomised across mean into ‘Poor’ and ‘Better’ categories. The frequency of tooth cleaning was categorised as once or less per day and twice or more per day. The status of use of chewing tobacco and smoking were categorised as never, former, and current. The habits were considered to be current if any participant has been using tobacco for one year or more and former if he/she has quit for one year or more. The frequency of dental visiting was categorised according to the responses to the question “When did you last visit a dentist?” as ‘Within previous one year’ and ‘Before previous one year/Never’. The outcome variable, SROH, was considered as a dichotomous variable (‘Poor’ and ‘Fair/Good/Very good’).

Statistical analysis was carried out using SPSS version 20. The univariate, bivariate, and multivariate analyses were performed for the outcome variable which was self-rated oral health. Clinical variables such as mean DMFT, mean number of sites with clinical attachment loss of ≥5 mm, and mean number of functional teeth were compared across the categories of SROH using ANOVA to test for statistical significance. For the bivariate and multivariable analyses, the outcome variable in the study was poor self-rated oral health. Categorically independent variables were compared across the outcome using cross-tabs and the Chi-square test. Unadjusted and adjusted odds ratios were estimated from binary logistic regression. A multivariable logistic regression model was built to identify risk indicators. The independent variables that showed *p* ≤ 0.2 at the bivariate level were all entered into the model simultaneously using ‘Enter method’. The confidence interval (CI) for all statistical analyses was 95%. Statistical significance between any two parameter estimates was determined based on the non-overlapping 95% CIs, while the statistical significance of effects as measured by Odds Ratios against references was determined if their 95% CI did not include unity. All the analysis was done using complex sampling plan in which sub-provinces were considered as strata and villages as clusters.

## 3. Results

The response rate in the present study was 62.3% considering the fact that 1401 eligible participants were approached. However, the intended sample size was 1160 participants of which a total of 873 participated from 50 villages, thus covering 75% of the calculated sample.

Table 1 shows the distribution of sociodemographic factors, habits, oral health behavior, and oral health status according to the SROH among the study population. The older the age group, the higher the proportion was of people rating their oral health as poor. Socioeconomic indicators such as lower levels of education, lower levels of income, and poor material circumstances were associated with poor SROH. People who had completed education of the secondary level (OR = 2.27, 95% CI = 1.32–3.88) and primary/less (OR = 4.09, 95% CI = 2.13–7.84) were more likely to rate their oral health as poor when compared to those who had completed post-secondary education. As the class of socioeconomic status determined by income lowered, the higher odds ratios were observed. Rural adults living under poor material circumstances showed higher odds (OR = 1.91, 95% CI = 1.19–3.07) for poor SROH than those living under better material circumstances. Current smokers exhibited higher odds for rating poor oral health. The second, third, and fourth quartiles of DMFT demonstrated higher odds ratio for poor SROH when compared to the first quartile of DMFT, exhibiting a dose–response-like relationship. Severe periodontal disease was found to be associated with poor SROH. 

Table 2 presents the proportion of people according to the rating of oral health and comparison of variables such as DMFT and the number of sites with clinical attachment loss of ≥5 mm according to categories of SROH. It was observed that the mean DMFT and the average number of sites with CAL of ≥5 mm was greatest for those rating poor oral health followed by those rating fair, good, and very good, in descending order.

Risk indicators associated with a poor rating of oral health are presented in Table 3. As seen from the multivariable logistic regression model, age, sex, monthly per capita income, smoking, DMFT, and periodontal disease were associated with poor SROH. Rural people in the age bracket of 40–44 years showed higher odds for rating their oral health as poor compared their younger and older (45–49 and 50–54 years) counterparts. Females were more likely to report poor oral health than males. Socioeconomic status lower than upper class (I) was observed to be significantly associated with poor SROH. Although the highest OR was seen for the lower class followed by the middle, lower-middle, and upper-middle classes, there was no statistical significance between the groups since the 95% CI overlapped. Smoking status was associated with the outcome. There was no difference between current and former smokers in rating their oral health as poor. People who visited a dentist in the last year showed higher odds (OR = 1.90, 95% CI = 1.11–3.26) for poor SROH compared to those who never visited a dentist or visited earlier than the last year. The higher the DMFT score, the higher the OR related to poor oral health was. A dose–response relationship was observed between the second, third, and fourth quartiles of DMFT and poor SROH. Severe periodontal disease was found to be associated with a poor rating of oral health.

## 4. Discussion

The present study aimed to investigate the poor self-rated oral health and its associations with socioeconomic conditions, clinical oral findings, and behavioural health factors in a rural Indian population. The prevalence of poor self-rated oral health was 15.2%, which was lower than in other populations from developed and developing countries [9,10]. The lower prevalence could be attributed to the difference in categorization of SROH as the outcome. Studies conducted among the Australian and Brazilian populations included fair and poor SROH [9,10,24] and the authors of this study considered only poor SROH as the outcome. In the present study, participants in older age groups of 40 years and above were more likely to rate poor oral health, which is quite similar to the findings observed in Guarani indigenous people [2]. It is consistent with the evidence that oral diseases are age-related. A multiple regression analysis showed that sex was associated with SROH after adjusting for other factors in the present study. Women are more conscious and are, therefore, more likely to rate their oral health negatively [5]. Previous studies have not found a significant association between sex and SROH though more female participants than male reported their oral health negatively [2,4].

Oral health exhibits inequalities across various socioeconomic measures. Lower levels of income showed a positive association with poor SROH. People in the upper-middle/middle class were found to report better oral health than people in the lower-middle and lower classes. The findings are similar to that of other studies conducted in countries such as Brazil, Australia, the United States, New Zealand, Canada, and Sweden [9,10,11,25]. Material circumstances and education did not show an association with SROH in the final adjusted model. Participants who did visit a dentist for oral health care within the last year were more likely to rate their oral health as poor. India is a developing country where many in rural areas have limited access to oral health care [16]. Oral health care services are mainly available at public hospitals and people in rural areas are unlikely to travel long distances to seek care at these hospitals with a long waiting time. Furthermore, the reimbursement of dental care through dental insurance is virtually non-existent and payment for oral health care services is mostly out of pocket. Due to this reason, people in rural areas only seek care when there are oral health problems affecting their functioning, thus rating poor oral health. The noted findings are quite similar to an Australian study where higher proportions of rural residents sought care when compared to their urban counterparts [26].

Smoking is a known risk factor for oral health. Smoking was found to be associated with poor SROH in the present study. Irrespective of the status, both former and current smokers were more likely to negatively rate their oral health compared to non-smokers. This is consistent with previous research [8,27]. Clinical oral conditions were found to be associated with poor SROH. Adults who had experienced dental caries (DMFT ≥ 4) were found to have poor SROH, and these findings were consistent with the study conducted by Bliznuik et al. where people with better oral health and those having a lower number of decayed teeth had better self-rated oral health [5]. The findings are similar to the evidence from the other studies that missing teeth and tooth loss are related to SROH [4,5]. The association between severe periodontal disease and poor oral health supports the findings from other studies [5,9]. Although the study was conducted in a developing country, many of the factors associated with poor oral health are similar to those found in developed countries.

This paper adds more information on self-rated oral health, which is a subjective measure and has been shown to be associated with sociodemographic and behavioural factors similar to objective measures, that is, clinical oral conditions. Moreover, poor SROH has been demonstrated to have clinical correlates. The present study could have focused on understanding the expectations of the rural populations, but this was not possible due to the paucity of data since it was collected for other studies. A common risk factor approach could be considered for future research with multi-dimensional self-rated measures and the use of oral health therapists for screening in rural areas [28].

## 5. Conclusions

The prevalence of poor oral health was high in the rural Indian adult population. Lower socioeconomic condition in terms of per-capita income, smoking, dental visiting within the last year, severe periodontal disease, and a higher number of dental caries experienced (DMFT ≥ 4) were associated with poor SROH. This signifies the plausibility of use of the global self-rating scale for oral health screening among rural populations in developing countries such as India.

## Figures and Tables

**Table 1:** Distribution of sociodemographic factors, habits, oral health status, and behaviour according to self-rated oral health in the study population and unadjusted association of various factors with poor self-rated oral health.

			Self-Rated Oral Health		
		N	Poor (*n* = 133) % (95% CI)	Very Good/Good/Fair (*n* = 740) % (95% CI)	Unadjusted OR (95% CI)
Age (years)	35–39	378	10.6 (7.1–15.4)	89.4 (84.6–92.9)	Ref
	40–44	147	19.0 (13.7–25.9)	81.0 (74.1–86.3)	1.99 (1.26–3.14) *
	45–49	152	19.1 (12.2–28.6)	80.9 (71.4–87.8)	1.99 (1.01–3.95) *
	50–54	196	18.4 (12.7–25.8)	81.6 (74.2–87.3)	1.90 (0.99–3.66)
Sex	Male	473	11.8 (9.3–14.9)	88.2 (85.1–90.7)	Ref
	Female	400	19.2 (14.9–24.5)	80.8 (75.5–85.1)	1.78 (1.21–2.60) *
Education	Post-secondary	162	6.8 (4.1–11.2)	93.2 (88.8–95.9)	Ref
	Secondary	466	14.2 (11.1–17.9)	85.8 (82.1–88.9)	2.27 (1.32–3.88) *
	Primary/less	244	23.0 (17.2–29.9)	77.0 (70.1–82.8)	4.09 (2.13–7.84) *
Income	Class I (Upper)	188	7.4 (4.5–12.2)	92.6 (87.8–95.5)	Ref
	Class II (Upper-Middle)	235	13.2 (9.0–18.9)	86.8 (81.1–91.0)	1.89 (1.02–3.52) *
	Class III (Middle)	153	18.3 (12.1–26.7)	81.7 (73.3–87.9)	2.78 (1.33–5.83) *
	Class IV (Lower-Middle)	164	19.5 (14.6–25.6)	80.5 (74.4–85.4)	3.01 (1.54–5.90) *
	Class V (Lower)	80	22.5 (14.1–33.9)	77.5 (66.1–85.9)	3.61 (1.64–7.96) *
Material circumstances	Better	343	10.5 (7.3–14.9)	89.5 (85.1–92.7)	Ref
	Poor	530	18.3 (14.8–22.4)	81.7 (77.6–85.2)	1.91 (1.19–3.07) *
Smoking status	Never	796	14.3 (11.6–17.5)	85.7 (82.5–88.4)	Ref
	Former	31	22.6 (12.3–37.7)	77.4 (62.3–87.7)	1.75 (0.83–3.69)
	Current	46	26.1 (14.9–41.6)	73.9 (58.4–85.1)	2.11 (1.07–4.17) *
Tobacco chewing status	Never	663	14.8 (11.7–18.5)	85.2 (81.5–88.3)	Ref
	Former	24	20.8 (8.7–42.1)	79.2 (57.9–91.3)	0.66 (0.22–1.95)
	Current	186	16.1 (11.0–23.1)	83.9 (76.9–89.0)	0.90 (0.55–1.50)
Frequency of tooth cleaning	Twice a day or more	368	14.4 (11.3–18.2)	85.6 (81.8–88.7)	Ref
	Once a day or less	505	15.8 (12.4–20.0)	84.2 (80.0–87.6)	1.12 (0.79–1.68)
Last dental visit	Within previous one year	612	13.6 (10.8–17.0)	86.4 (83.0–89.2)	Ref
	More than previous one year/Never	261	19.2 (13.9–25.9)	80.8 (74.1–86.1)	1.51 (0.96–2.38)
DMFT ǂ	Q1 (DMFT = 0–3)	220	5.9 (3.7–9.2)	94.1 (90.8–96.3)	Ref
	Q2 (DMFT = 4–7)	219	14.2 (9.9–19.8)	85.8 (80.2–90.1)	2.63 (1.65–4.17) *
	Q3 (DMFT = 8–12)	209	17.7 (12.7–24.1)	82.3 (75.9–87.3)	3.43 (2.04–5.76) *
	Q4 (DMFT ≥ 13)	225	23.1 (17.8–29.4)	76.9 (70.6–82.2)	4.79 (2.81–8.15) *
Periodontal disease	No/Mild	464	11.2 (8.1–15.2)	88.8 (84.8–91.9)	Ref
	Moderate	313	16.0 (11.4–21.9)	84.0 (78.1–88.6)	1.51 (0.93–2.43)
	Severe	92	30.4 (20.5–42.6)	69.6 (57.4–79.5)	3.47 (1.79–6.72) *

ǂ Decayed Missing and Filled Teeth; OR—Odds ratio; CI—Confidence interval; * odds ratio not including unity indicates statistical significance.

**Table 2:** Distribution of clinical oral health variables according to self-rated oral health.

Self-Rated Oral Health	Very Good	Good	Fair	Poor	*p* Value
*n* (%)	6 (0.7)	236 (27.0)	498 (57.0)	133 (15.2)	
DMFT ǂ µ (±SD)	7.0 (±4.64)	5.63 (±5.17)	9.57 (±6.71)	11.73 (±7.16)	<0.001 *
Number sites with clinical attachment loss of ≥5 mm µ (±SD)	0.67 (±1.03)	0.96 (±2.51)	1.01 (±2.44)	2.07 (±3.44)	<0.001 *

ǂ Decayed Missing and Filled Teeth; * *p* value < 0.001 statistically significant from ANOVA. µ-mean; SD—Standard deviation.

**Table 3:** Multivariable logistic regression model ^†^ evaluating various risk indicators of poor self-rated oral health among adults in rural India.

		Adjusted OR (95% CI)
Age (years)	35–39	Ref
	40–44	2.03 (1.18–3.49) *
	45–49	1.89 (0.96–3.71)
	50–54	1.47 (0.65–3.30)
Sex	Male	Ref
	Female	2.22 (1.36–3.63) *
Education	Post-secondary	Ref
	Secondary	1.36 (0.71–2.58)
	Primary/less	1.78 (0.89–3.57)
Income	Class I (Upper)	Ref
	Class II (Upper-Middle)	2.10 (1.02–4.32) *
	Class III (Middle)	2.93 (1.30–6.62) *
	Class IV (Lower-Middle)	2.84 (1.18–6.82) *
	Class V (Lower)	3.48 (1.34–8.96) *
Material circumstances	Better	Ref
	Poor	0.97 (0.54–1.77)
Smoking status	Never	Ref
	Former	2.05 (1.01–4.13) *
	Current	2.14 (1.01–4.52) *
Last dental visit	Within previous one year	1.90 (1.11–3.26) *
	More than previous one year/Never	Ref
DMFT ǂ	Q1 (DMFT = 0–3)	Ref
	Q2 (DMFT = 4–7)	2.77 (1.74–4.40) *
	Q3 (DMFT = 8–12)	3.14 (1.74–5.64) *
	Q4 (DMFT ≥ 13)	4.22 (2.32–7.68) *
Periodontal disease	No/Mild	Ref
	Moderate	1.31 (0.69–2.48)
	Severe	2.93 (1.39–6.19) *

Coefficient of determination for the multivariable logistic regression model—McFadden’s Pseudo R^2^ = 0.134 and Nagelkerke’s Pseudo R^2^ = 0.188; † Multivariable logistic regression model built for the outcome variable SROH (Binary) by including all the explanatory variables shown in the table using forced entry method. B—Partial regression coefficients; S.E—Standard error; ǂ Decayed Missing and Filled Teeth; OR—Odds Ratio; CI—Confidence Interval; * odds ratio not including unity indicates statistical significance.
